# DOCKSCORE: a webserver for ranking protein-protein docked poses

**DOI:** 10.1186/s12859-015-0572-6

**Published:** 2015-04-24

**Authors:** Sony Malhotra, Oommen K Mathew, Ramanathan Sowdhamini

**Affiliations:** National Centre for Biological Sciences (TIFR), UAS-GKVK Campus, Bellary Road, Bangalore, 560 065 India; SASTRA University, Tirumalaisamudram, Thanjavur, 613 401 Tamil Nadu India

**Keywords:** Docking, Interactome, Protein interfaces, Quaternary structure prediction

## Abstract

**Background:**

Proteins interact with a variety of other molecules such as nucleic acids, small molecules and other proteins inside the cell. Structure-determination of protein-protein complexes is challenging due to several reasons such as the large molecular weights of these macromolecular complexes, their dynamic nature, difficulty in purification and sample preparation. Computational docking permits an early understanding of the feasibility and mode of protein-protein interactions. However, docking algorithms propose a number of solutions and it is a challenging task to select the native or near native pose(s) from this pool. DockScore is an objective scoring scheme that can be used to rank protein-protein docked poses. It considers several interface parameters, namely, surface area, evolutionary conservation, hydrophobicity, short contacts and spatial clustering at the interface for scoring.

**Results:**

We have implemented DockScore in form of a webserver for its use by the scientific community. DockScore webserver can be employed, subsequent to docking, to perform scoring of the docked solutions, starting from multiple poses as inputs. The results, on scores and ranks for all the poses, can be downloaded as a csv file and graphical view of the interface of best ranking poses is possible.

**Conclusions:**

The webserver for DockScore is made freely available for the scientific community at: http://caps.ncbs.res.in/dockscore/.

**Electronic supplementary material:**

The online version of this article (doi:10.1186/s12859-015-0572-6) contains supplementary material, which is available to authorized users.

## Background

Proteins in the cell rarely act in isolation and in fact, are known to interact with other biomolecules like DNA, RNA, other proteins, small molecules *etc.* [1]. Studying and understanding these interactions will provide insights into the physiological roles and regulation mechanism. These interaction sites can further be studied for the effect of mutations or for therapeutic purposes. There are excellent experimental methods available to study protein-protein interactions (like yeast two-hybrid, co-immunoprecipitation *etc.* [2,3]) and also to pinpoint the site of interactions using mutation studies, structure determination methods (such as X-ray, NMR) and label transfer [4]*.* Protein-protein docking is the computational method to study protein-protein interactions, based on electrostatics, shape and geometric complementarities [5-8]. Docking of the interacting pairs of proteins provides insights into the specific atomic details of interactions. There are several docking programs available as downloadable softwares and as webservers (such as HADDOCK, [6]; ZDOCK, [9]; ClusPro, [10]; GRAMM-X webserver, [11]; FRODOCK, [12] and HADDOCK webserver [13]). These programs employ scoring functions which are based on ranking the poses based on the energy values. However, upon docking, there are number of proposed solutions and selection of biologically meaningful pose from this pool still remains a challenging task [14].

It is possible to limit the search space by guiding the docking around certain residues based on evolutionary or biochemical data. However, in the absence of such an information or even from a set of docking decoys, selection of the best docked pose becomes a difficult task. In these cases, one can analyze the interfaces which are proposed by the docking program. We had recently proposed a scoring scheme, named DockScore, to re-rank the docked poses and identify the native or near-native poses from the pool [15]. DockScore is initiated with the identification of interface residues based on distance-based criteria and then considers several interface parameters namely surface area, conservation, hydrophobicity, spatial clustering and short contacts to perform the scoring.

We had assessed the performance of DockScore on 30 protein-protein complexes and CAPRI targets and compared the performance of our scoring scheme with two other methods namely dDFIRE [16] and FireDock [17] for our testing dataset. We have shown that DockScore was able to rank the native complex as a top-ranking pose in 26 out of the 30 complexes tested, whereas dDFIRE and FireDock were able to achieve this in 16 of the cases [18].

There are several scoring programs available as a downloadable package [19-21] in order to re-rank the docked poses, but the webserver implementation or availability for easy access is less common [17,22,23]. In this article, we report the availability of DockScore in the public domain as a webserver for the scientific community. This includes user-interactive tools webserver and convenient graphical display of interface regions of high-scoring poses. The webserver can also be used to perform scoring of protein-protein interactions or re-ranking the docked poses to identify the biologically meaningful pose(s) out of the pool. Users can upload a zipped file containing the pool of docked poses which need to be ranked. Each parameter of the scoring scheme can be turned on/off depending on the discretion of the user. In the output, we provide a list of all docked poses with all the scores marked in the list. User can also visualise the five top-most poses in Jmol with the interface residues from two protein chains colored differently. The file containing different scores and ranks of the docked poses can be downloaded in CSV format.

## Implementation

### DockScore webserver parameters

The webserver presented here employs the scoring scheme called DockScore to perform the ranking of the docked poses. It utilizes the parameters of the interface formed upon interaction of the two given protein chains. These interface parameters are surface area, conserved residues, hydrophobicity, short contacts and spatial clustering. There is an additional parameter, which is based on the presence of positively charged residues at the interface. This can be employed selectively and especially when the interacting protein chains are DNA-binding (*for e.g.* transcription factors) or RNA-binding in nature. The presence of positively charged residues at the interface is penalized to minimize the overlap of protein-protein interaction site with that of DNA-binding region.

The interface residues are identified using the distance-based criteria, inter-chain C^β^-C^β^ distance cut-off of 7 Å. The interface parameters that are employed for performing the ranking are explained below briefly. Weights for each of the parameter can be easily assigned, if a new training dataset is choosen by estimating the importance of each parameter (*i.e.* using only one parameter at a time and assessing the performance, please refer to DockScore publication [18]) Each of these parameters is assigned weights based on the training dataset.

**Figure 1:**
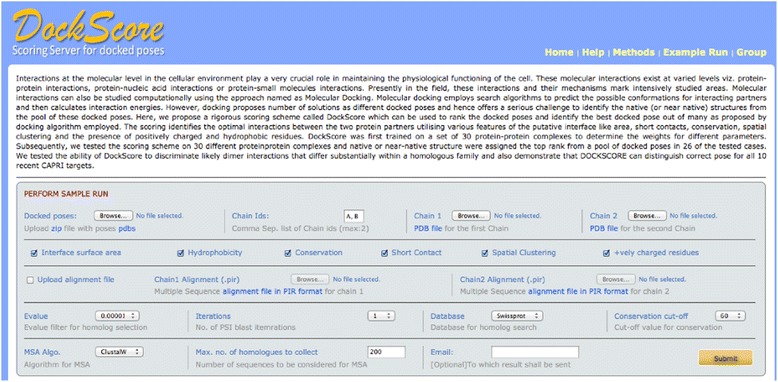
The methodology and parameters of the webserver. All the input options are listed and a link for sample run is provided. Link for help page and methods behind the webserver are also provided. Different parameters which are considered for performing scoring: The user can select the parameters to be used for scoring. For assessing the conservation of interface residues, user can select the parameters for collection of homologs and the conservation score cut-off can be set to 60 if close homologues are employed, however when distant homologues are included in the alignment, this threshold should be set to 40.

The parameters, which are used for the scoring, are explained briefly (Figure 1) and for details regarding each parameter, please refer the DockScore publication [18] and the webserver help page.

1. Surface Area: It is computed using NACCESS ('NACCESS', computer program. (1993) by S. J. Hubbard, J. M. Thornton).
2. Conservation of residues: The individual protein chains are used as queries to perform PSI-BLAST [24] in order to collect homologues from the SWISSPROT database and multiple sequence alignment is built using CLUSTALW [25,26]. Conservation scores per residue are evaluated using our *in-house* program MOTIFS [27], where permitted amino acid exchanges and identities are scored high. The score cut-off of 60 is usually used for close homologues and 40 if the distantly related members are included in the alignment, to identify the conserved residues (Figure 1). The number of conserved residues at the interface is normalized by the total number of interface residues.
3. Inter-chain short contacts: Our in-house program CoilCheck [28] is employed to identify short contacts.
4. Spatial Clustering: The pairwise distances between the interface residues were computed between the two chains and the residues with a C^β^-C^β^ distance cut-off of 14 Å were considered as spatially clustered residues.
5. Hydrophobic residues: We ranked those docked poses with high numbers of hydrophobic residues (A, V, L, I, M, F, W and Y) at the interface with a high score, as protein-protein interfaces are known to be rich in such residues [1,29,30].

### Input files

**Figure 2:**
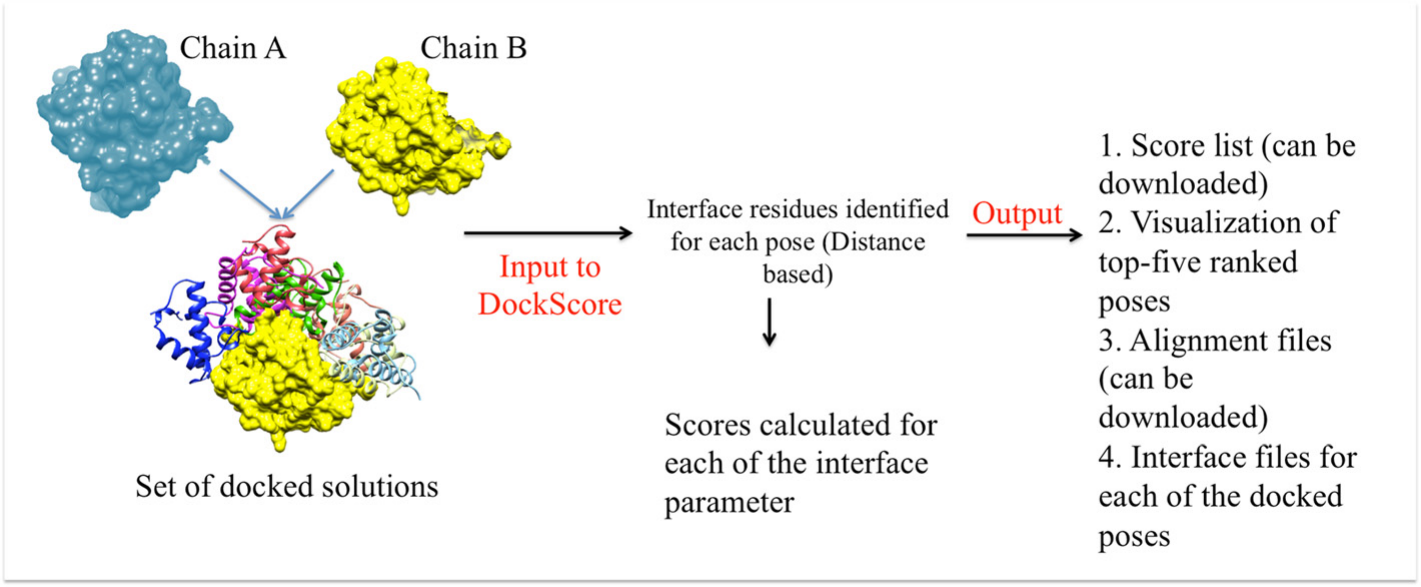
The workflow of the webserver. Input options: User can upload a zipped file containing all the docked poses to be ranked and the coordinate files for both the protein chains. Different parameters which are considered for performing scoring: The user can select the parameters to be used for scoring. For assessing the conservation of interface residues, user can select the parameters for collection of homologs. Main output options: The five top-ranking poses can be visualised with Jmol and the list with the scores can be downloaded in CSV format.

For performing scoring, the following files should be supplied as an input to the server (Figure 2):

1. Zipped file containing the docked poses in PDB format with coordinates of both the interacting chains
2. PDB coordinates and chain ID of both the protein chains used to perform docking

The computation is not initiated if the files are not in PDB format and coordinates for both the interacting chains are not provided by the user.

### Output details

After performing scoring, the following scores are reported:

1. Scores for each of the parameters individually
2. Average score

All parameters are assigned an equal weight of 1 and the average is reported for each docked pose.

3. Normalized weighted score

The weights derived were further normalized by the total sum of weights and normalized weighted score was calculated.

4. Z-score for the normalized weighted score

For each pose, a Z-score is calculated to assign a significance of normalized weighted score. We have tested this on our test dataset (30 cases) and note Z-score >1.5 is discriminatory to identify the native (or near-native) pose (Additional file 1).

In the output page, webserver displays a list as an output with docked poses ranked according to the normalized weighted score (Figure 2). The list can be sorted according to any of the parameter/score by clicking on that column. This list with the entire scores can also be downloaded from the webserver in the CSV format. The user can input his/her email ID and the result link will be posted at this address.

Subsequent to the scoring, the five top-most poses can be visualized using JSmol (JSmol: an open-source Java viewer for chemical structures in 3D. http://wiki.jmol.org/index.php/JSmol). The interface residues from the two interacting chains are highlighted in different colors (Figure 2).

## Results and discussion

The server can be used for performing the scoring of protein-protein interactions. Figure 1 represents the screenshot of the server explaining all the parameters considered for scoring. The user has a choice to select parameters to be employed for scoring, or the user can rank the poses based on any parameter or normalized score of their choice upon scoring.

### Framework

This website is in the public domain and is open to all users and there is no login requirement.

In the output page, webserver displays a list with docked poses ranked according to the normalized weighted score. The list can be sorted according to any of the parameter/score by clicking on that column. This list with the entire scores can also be downloaded from the webserver in the CSV format. The user can input his/her email ID and the result link will be posted at this address.

**Figure 3:**
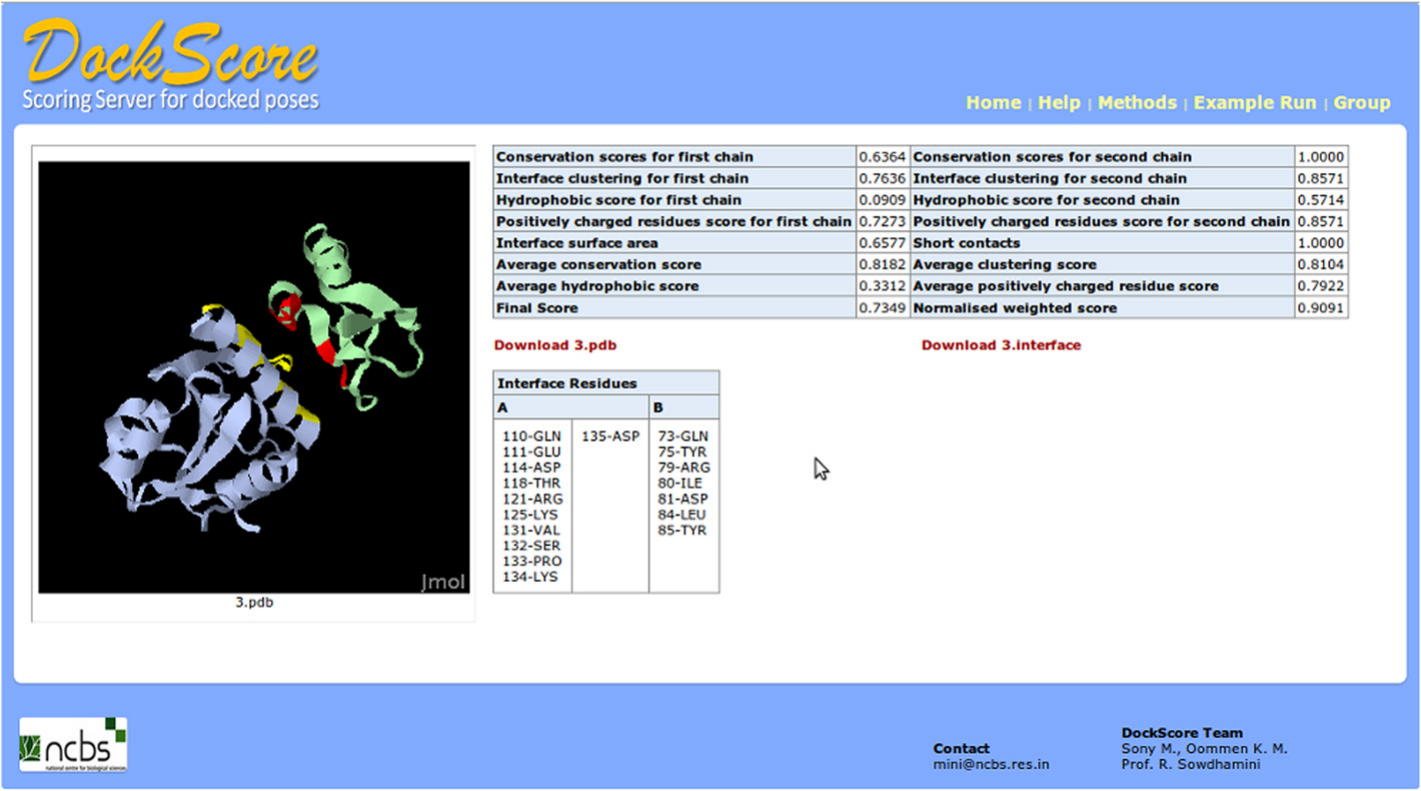
An example output for individual pose. For all the docked poses uploaded on the webserver, a link is provided for viewing the interface residues and the result for each of the interface parameter.

For an example output, it takes 70 seconds to rank 100 poses (7 sec/docked pose), for a homodimer with 73 residues on the webserver with 8 cores; Intel(R) Xeon(R) CPU E5620 @ 2.40GHz and 5Gb RAM to finish the computation and obtain the results. For all the docked poses, a link is provided to view the interface residues and the results for each of the parameter (Figure 3). Subsequent to the scoring, the five top-most poses can be visualized using Jmol (Jmol: an open-source Java viewer for chemical structures in 3D. http://www.jmol.org/). The interface residues from the two interacting chains are highlighted in different colors.

### Test example

**Figure 4:**
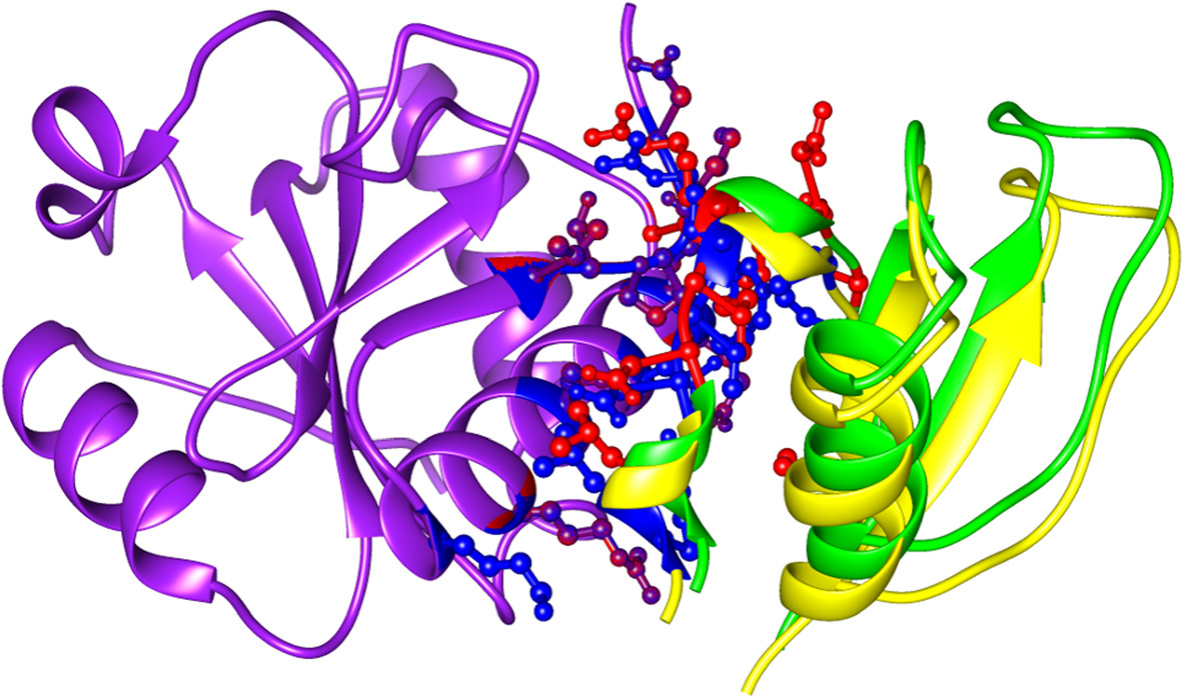
Interface analysis of a CAPRI target. For 1SYX, one of the chains is superposed for native and the pose ranked next to native. In yellow is the second protein chain from the native complex and blue represents the second chain of the pose ranked second. The interfaces were very similar in both the poses (Interface residues are represented as ball and sticks. In blue are the interface residues from the native complex and red are the interface residues from the second pose).

DOCKSCORE is shown to work very well earlier [18]. However, as an example, we highlight the case study which is one of the CAPRI-8 targets (corresponding to PDB code: 1SYX). 1SYX is a crystal structure highlighting the interactions between full-length protein U5-15 K and GYF-domain of U5-52 K [31]. We performed docking between these two individual proteins using FRODOCK [12] to obtain 99 poses, and the native complex structure was added to this pool of poses. This pool was submitted to the webserver and the docked poses were ranked. The native complex was ranked first and the pose ranked second was structurally very similar to the native complex structure (Figure 4). The overlap among interface residues was 90% and 86% for the two chains (Table 1). The scores and fraction of overlap between the interface residues (with native) are provided as Additional file 2.

**Table 1:** **Interface analysis**

**Chain A**		**Chain B**	
**Native**	**Ranked next to native**	**Native**	**Ranked next to native**
Q110	D108	Q73	P69
E111	Q110*	Y75	Q73*
D114	E111*	R79	Y75*
T118	T118*	I80	N76
R121	R121*	D81	R79*
K125	V131*	L84	I80*
V131	S132*	Y85	D81*
S132	P133*		L84*
P133	K134*		
K134	D135*		
D135			

In the example of 1SYX, the native complex was ranked as the first pose using DockScore. The pose ranked next to the native complex was structurally very similar to the native pose and the interface residues were overlapping with the native complex interface residues (marked in asterisk).

*Overlapping interface residues between native and the pose ranked second.

### Scale-up in docked poses

We next examined the effect of sampling additional number of docked poses, rather than 99 poses, with two cases referred as ‘example’ and a ‘difficult example’ derived from the DockScore testing dataset.

In the ‘example’ (PDB code 1GHD), which was a success while testing DockScore, we sampled 1000 docked poses to see if DockScore is still able to rank the native pose as a top-ranking pose out of a pool of 1000 docked poses. We find that the performance of DockScore is not reduced due to enhanced sampling (Additional file 3). In the ‘difficult example’ (PDB code 1IZY), the native pose was not the top-ranking pose while performing the test runs. So, we sampled 1000 poses to see if DockScore ranks the native pose as top-ranking one and still the performance did not seem to improve (Additional file 4).

## Conclusions

DockScore helps in distinguishing the native/near-native complexes from the pool of docked poses. It can be employed post-docking to rank the poses. Different interface parameters are considered to perform this scoring like interface surface area, conservation, hydrophobicity, spatial clustering and short contacts. We implemented this scoring scheme in the form of webserver for its use by the community. The web tool provides a list of all scores for the given docked poses provided as input. The top-ranking poses can also be visualized.

## Availability and requirements

- **Project name:** DockScore webserver
- **Project home page:**http://caps.ncbs.res.in/dockscore/
- **Operating system(s):** Platform independent
- **Programming language:** Perl, Java, JavaScript
- **Other requirements:** Java plug-in for the respective browser
- **License:** Free for academic use
- **Any restrictions to use by non-academics:** Free for academic purposes. For commercial use please contact the corresponding author

The software driving the webserver can be made available upon request for academic use.

## Additional files

Additional file 1:
**Z-scores for the test cases.** The Z-scores for all the docked poses and native pose is calculated for the normalized weighted score. The scores for native pose is plotted as filled circles. Additional file 2:
**Scores for the test example 1SYX.** The scores for each of the docked poses are listed and the last column highlights the fraction overlap of interface residues with the native pose. 100.pdb (marked in bold) is the native complex. Additional file 3:
**Large scale docking for a success case.** The list provides the scores for 1000 poses of a complex 1GHD (the native pose was the top-ranking pose while performing the test runs with 100 poses). Sheet 1 has results for 100 poses and sheet 2 has results for 1000 poses and the native poses (100.pdb and 1000.pdb respectively) are marked in bold. Additional file 4:
**Large scale docking for a failed case.** The list provides the scores for 1000 poses of a complex 1IZY (the native pose was not the top-ranking pose while performing the test runs with 100 poses). Sheet 1 has results for 100 poses and sheet 2 has results for 1000 poses and the native poses (100.pdb and 1000.pdb respectively) are marked in bold.
