# Supplementary figures and images for: DOCKSCORE: a webserver for ranking protein-protein docked poses
Source: BMC Bioinformatics. 2015 Apr 24;16(1):127. doi: 10.1186/s12859-015-0572-6 (PMC4414291; doi:10.1186/s12859-015-0572-6)

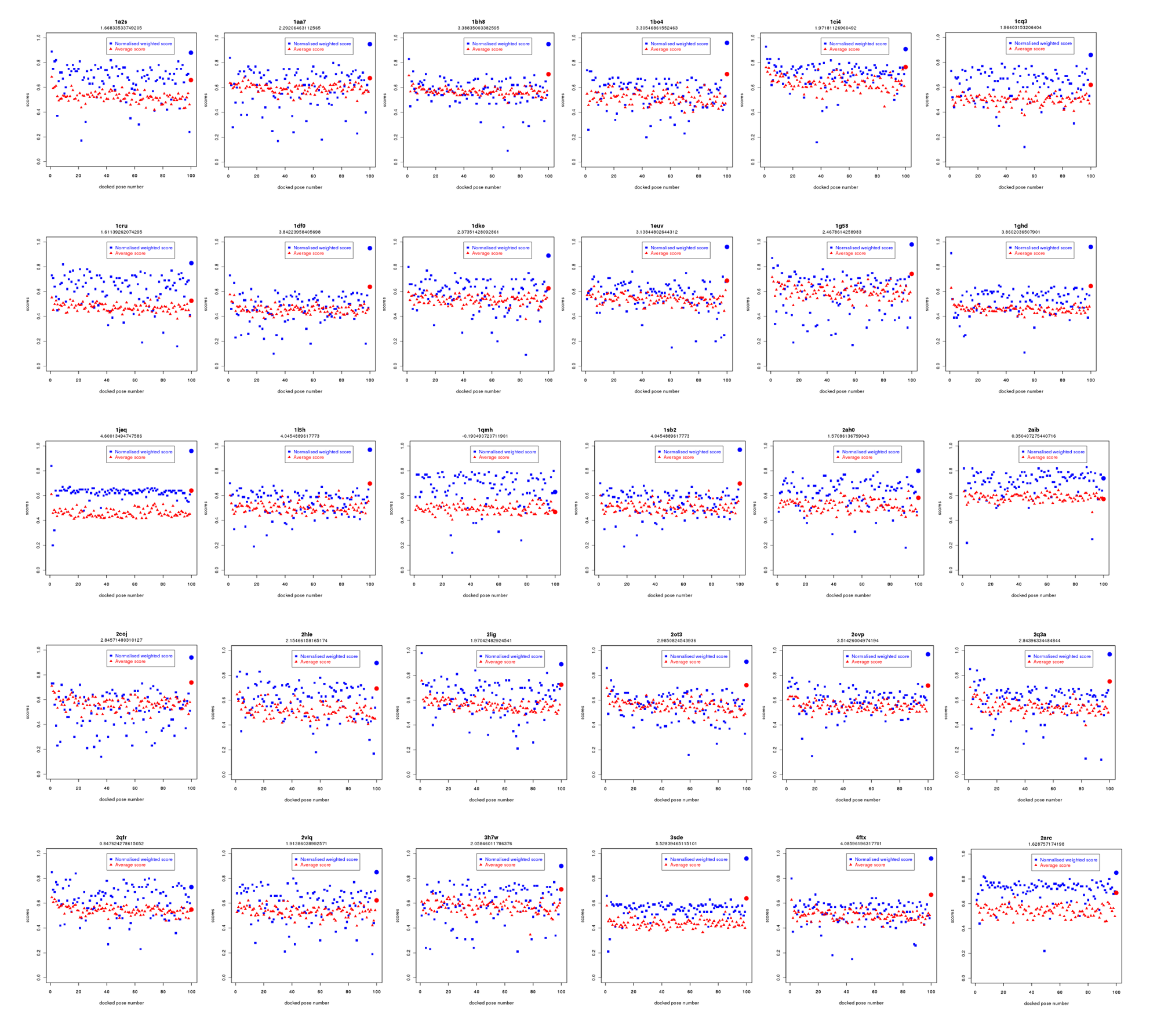

Supplement: Additional file 1: — Z-scores for the test cases. The Z-scores for all the docked poses and native pose is calculated for the normalized weighted score. The scores for native pose is plotted as filled circles. [file 12859_2015_572_MOESM1_ESM.tiff]
